# Polyphenol Extracts from Red Wine and Grapevine: Potential Effects on Cancers

**DOI:** 10.3390/diseases6040106

**Published:** 2018-11-18

**Authors:** Souheila Amor, Pauline Châlons, Virginie Aires, Dominique Delmas

**Affiliations:** 1 Université de Bourgogne-Franche Comté, Dijon F-21000, France; souheila.amor@u-bourgogne.fr (S.A.); paulinechalons@orange.fr (P.C.); virginie.aires02@u-bourgogne.fr (V.A.); 2 Centre de Recherche INSERM U1231-Cancer and Adaptative Immune Response Team–Bioactive Molecules and Health research group, Dijon F-21000, France

**Keywords:** red wine, polyphenols, cancers, colorectal

## Abstract

Wine has been popular worldwide for many centuries and currently remains an important component of our diet. Scientific interest in wine and its health effects has grown considerably since the 1990s with the emergence of the “French Paradox” concept, correlating moderate wine consumption, a characteristic of the Mediterranean diet, and low incidence of coronary heart diseases. Since then, the positive effects on health, health promotion, disease prevention, and disease prognosis of moderate wine consumption, in particular red wine, have been attributed to its polyphenolic compounds such as resveratrol, quercetin, and other flavonoids acting as antioxidants. Several epidemiological, in vivo and in vitro, studies have reported that moderate red wine or red wine polyphenolic extract consumption may be active in the prevention and treatment of chronic diseases such as cardiovascular disease, metabolic syndrome, degenerative pathologies, and cancer. The aim of this review is to summarize the current findings about the effects of red wine polyphenols on cancer and to discuss how the polyphenolic composition of red wine may influence its chemopreventive properties.

## 1. Introduction

Wine has been produced since the beginnings of civilization, presumably starting in the Near East; traces are found in the Egyptian hieroglyphs, the code of the Babylonian king Hammurabi, and in Assyrian bas-reliefs. The influence of wine on the development of most Eurasian societies is considerable and it is the only food for which such a sacred and symbolic character has been attributed. Since antiquity, wine has been an important part of the diet of most countries around the Mediterranean. It is consumed in several forms, and it is also a prime commercial activity. Today, wine consumption, even though it has been decreasing over the past several decades, remains important in the countries of the Mediterranean basin, and wine continues to appear on our tables by remaining a central element of our diet and culture. It should be noted that consumption is increasing in emerging countries (China, Brazil, Argentina) and in other industrialized countries (USA, Japan).

Also in this context, which combines popular beliefs, economic issues, and health issues both in terms of prevention with respect to the population at risk (i.e., the presence of alcohol), and in terms of its beneficial effects on health due to the presence of many bioactive molecules, it is important to provide a scientific rationale for moderate wine consumption in order to establish an effective public health policy and to identify new bioactive molecules in grapes that can have an advantage in a healthcare strategy.

During the last decade, numerous studies have revealed that a moderate consumption of wine, as part of a healthy diet, is associated with protective effects against relevant chronic diseases despite its ethanol content and it harmful effects. For several years, numerous epidemiological studies have maintained that a moderate consumption of wine lowered the risks of mortality due to coronary diseases, compared to wine abstinence [1,2]. For example, in France, as compared with other western countries with a fat-containing diet, the strikingly low incidence of coronary heart diseases is partly attributed to the moderate consumption of red wine [3]. This is how what was commonly called the “French paradox” was born. Nevertheless, since the 1990s, this term has been controversial and seemed derogatory, the French paradox likely resulting more from a Mediterranean-type diet [4,5,6].

Indeed, several international long-term studies, sponsored by the World Health Organization (WHO), such as the MONICA (monitoring of trends and determinants in cardiovascular disease) study during the 1980s and more recently the PREDIMED (Prevención con Dieta Mediterrénea) Project, revealed the health benefits of wine that was associated with a diet rich in fruits, vegetables, and olive oil, commonly called the Mediterranean diet. The MONICA study has shown that in France, as compared with other western countries (such as the UK or the US), despite a fat-containing diet, a strikingly low incidence of coronary heart diseases was observed and is partly attributed to the moderate consumption of red wine [7,8,9]. The Mediterranean diet can be described as abundant in plant-based foods such as whole grains, legumes, seeds, fruits, and vegetables, with olive oil the main source of dietary fat; a limited intake of red and processed meat, favoring a low to moderate intake of low-fat dairy and a moderate consumption of fish; and emphasizing regular, but moderate, alcohol (mostly red wine) consumption with meals [10]. The Mediterranean diet has thus gained strong scientific support for providing protection against relevant chronic diseases such as cardiovascular diseases (CVDs), diabetes, as well as protection against some cancers [11,12]. These observations have aroused increasing interest in the scientific community in understanding the underlying mechanisms involved. In view of these epidemiological results, it seems essential to standardize studies to describe the effects resulting from a consumption of red wine alone. In this way, to study the potential effect of red wine on cardiovascular events, we have shown an amelioration of blood parameters (decrease in total cholesterol andlow density lipoprotein, LDL); increase in erythrocyte membrane fluidity and antioxidant status) in a group of selected post-myocardial infarct patients receiving 250 mL/day of red wine for 2 weeks, in comparison to patients receiving water in a controlled environment in the hospital [13]. For the first time in a controlled environment, these results reinforced the idea that a moderate consumption of red wine, even for a short period, associated with a ‘‘Western prudent’’ diet, improves various blood parameters in the lipid and antioxidative status in patients with previous coronary ischemic accidents.

**Figure 1:**
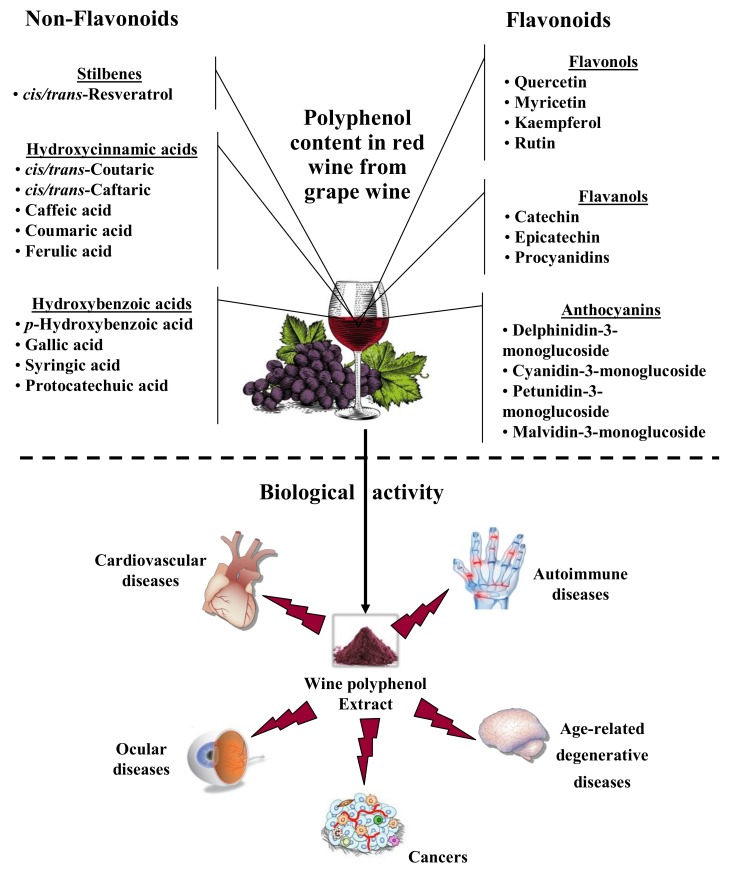
Major constituents in red wine from grapes and the potential biological effects against various diseases.

Other studies tend to show beneficial effects related to wine consumption on the occurrence of degenerative pathologies, for example, age-related macular degeneration (AMD) [14], dementia [15,16,17,18], and cancers [19,20]. Subsequently, various studies have been conducted to determine the effect of different preparations enriched with wine polyphenol or polyphenol grape extracts on various pathologies such as cardiovascular [21,22], ocular [23], inflammatory and age-related degenerative diseases [24,25], and cancers [26,27] (Figure 1).

## 2. Wine and Cancers

### 2.1. Wine Composition and Variability of Effects

Cancer is one of the major causes of death in the world and is responsible for an estimated 9.6 million deaths in 2018. The most common cancers equally affect the lung and breast, with 2.09 million cases, colon (1.80 million cases), prostate (1.28 million cases), skin (non-melanoma) (1.04 million cases), and stomach (1.03 million cases). Tobacco use, alcohol abuse, an unhealthy diet, and physical inactivity are described as major cancer risk factors worldwide and modifying or avoiding these key risk factors can significantly reduce (30%–50%) the burden of cancer [28,29]. Therefore, the strong interrelationship between nutrition and diet and the occurrence of tumors is abundantly described in the literature [30], and it is observed that a plant-rich diet may prevent 30%–40% of all cancer types [31,32,33]. Also, high intake of dietary fiber from fruit, vegetables, and whole grains is inversely associated with colorectal cancer risk [34]. Such benefits have been partly attributed to the presence of significant amounts of phenolic antioxidants in fruits and vegetables and are believed to contribute to their chemopreventive effects, notably in the colon [35,36,37]. Therefore, several studies (in vitro and/or in vivo) have demonstrated the anticancer potential of wine phenols (i.e., resveratrol, quercetin, (+)-catechin). In this way, various case–control studies have shown that a moderate red wine consumption exerts a protective effect on colorectal cancer in both men and women [19,20]. Moreover, other case–control studies have examined the association between wine and the Mediterranean diet, showing a lower risk of colon cancer and certain other cancers, such as urinary tract tumors, compared to other diets [38,39,40]. Nevertheless, one study did not find an inverse association between moderate red wine intake and the risk of colorectal cancer [41] or breast cancer [42]. This controversy may result from the amount and quality of polyphenols present in red wine. Indeed, red wine contains a range of biologically active polyphenols, including phenolic acids, trihydroxystilbenes, and flavonoids (Figure 1). In previous studies, we have shown both that a mixture of polyphenol extract from vine shoots demonstrates more antiproliferative activity on colon cancer cells than resveratrol alone, due to a synergism between polyphenols [43], and that the quantity and quality of the polyphenols present in wine also played an important role. 

Wine composition is a complex and unique combination dependent on various factors such as the vine, the climate, the country, and the year. Thus, the amount of polyphenols in wine, although varying greatly, is estimated to be around 190–290 mg/L in white wines and 900–2500 mg/L in red wines [44,45]. This variability of the polyphenol composition seems very important in determining its effects. We demonstrated in a previous study that the lengthening of the maceration time modified and enriched the polyphenol composition of red wine [26]. In comparison with red wine extracts, whose maceration time was less and therefore whose polyphenol composition was lower, the extract resulting from a longer maceration in red wine showed a more pronounced antiproliferative effect, with respect to the colonic cancer lines tested [26]. The quantitative aspect is not the only important parameter; the qualitative composition of the wines is also a crucial factor in the observation of the beneficial effects or their absence. Very interestingly, some polyphenols do not act in a synergistic manner but rather in an additive manner and in some cases have an opposite effect [26]. These data raise the crucial role of the polyphenol composition of wine where an imbalance between polyphenolic species may increase or conversely reduce their beneficial effects. The presence of (+)-catechin reduces the synergism effect between resveratrol and quercetin, which could explain the differences studies have shown in colon cancer risk reduction with moderate red wine consumption in humans [19,20], or in animal models [27,46], while others showed no effect [41,47]. It therefore seems essential to study the effects of a wine in relation to its phenolic composition. Some of these polyphenols present in large quantities have a strong activity when they are studied separately. This is particularly the case for the most well-known resveratrol [48,49], quercetin [50], (+)-catechin [51], and gallic acid [52,53], which present a variety of chemopreventive properties.

### 2.2. Wine and Colorectal Cancer

Consequently, the effects of wine consumption, particularly in a healthy population, may depend on the composition of the wine or grape polyphenols and their bioavailability [54,55]. Otherwise, dietary polyphenols exert a beneficial effect at a local level (colon) directly, during their passage through the oral cavity and the gastrointestinal tract, and at a systemic level, after being absorbed. Therefore, one of the organs that can be a targeted is the intestine and colon. 

**Figure 2:**
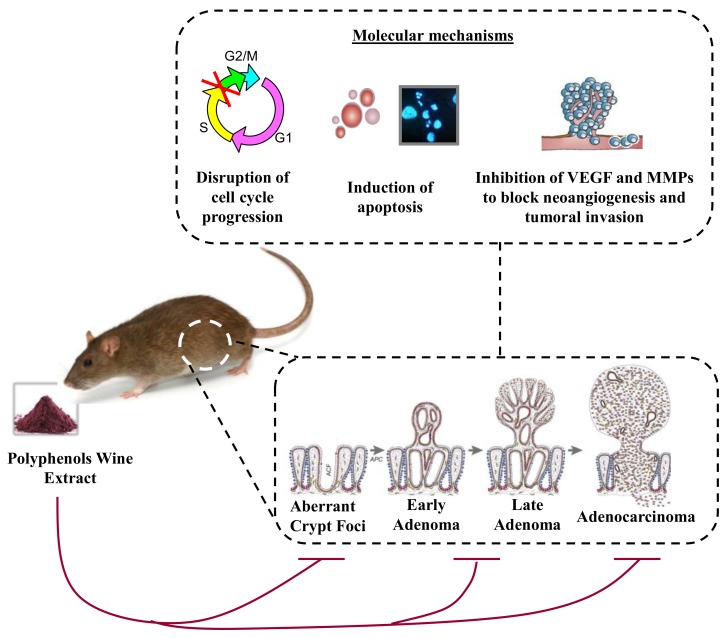
Wine polyphenol extract is able to prevent aberrant crypt foci formation in various animal models (mice, rats), which is the first step of colorectal carcinogenesis, and can block the different steps leading to adenocarcinoma development. The effects involved different molecular mechanisms such as an arrest of the cell cycle in the S phase, an induction of apoptosis through caspase activation, and an inhibition of angiogenesis and tumoral invasion through a decrease in vascular endothelial growth factor (VEGF) secretion and matrix metalloproteinases (MMP) activities.

Colorectal cancer (CRC) is the third most common form of cancer occurring worldwide. A total of 1.8 million cases each year are recorded [28]. Epidemiological and experimental studies have shown that the risk factors of developing colon cancer can be attributed mostly to multifactorial environmental factors. Therefore, the majority (95%) of CRC diagnoses begin as noncancerous polyps of the intestinal epithelium on the inner lining of the colon or rectum that have accumulated oncogenic mutations over time. Noncancerous polyps may become malignant and transform into adenomatous polyps if left undetected. Progression through the various stages of the adenoma–carcinoma process is significantly influenced by environmental factors inherent in the western lifestyle, such as the diet, the sedentary lifestyle, as well as smoking and the consumption of alcohol. Indeed, the consumption of high levels of red meat and fat together with low levels of fruits and vegetables has been suggested to increase the risk of CRC [56]. Several studies indicate that the establishment of nutritional prevention could significantly reduce the occurrence of colon cancer. As documented in the literature, the benefits of the Mediterranean diet include protection against cardiovascular disease, metabolic diseases such as diabetes, obesity, and various cancers, and now is a recommended diet, among others, in strategies for cancer prevention. However, the mechanisms involved in the protection against CRC by patients following the Mediterranean diet are not completely identified and understood. For more insight on the Mediterranean diet, Donovan et al. have reviewed preclinical and clinical studies conducted over the last 10 years on the impact of a Mediterranean diet-eating pattern [57]. New cancer prevention strategies include a number of dietary constituents described as promising chemopreventive agents [58]. Chemoprevention is defined as the use of specific natural products or synthetic chemical agents to delay, prevent, or reverse lesions before the development of invasive cancer [59]. Otherwise, previous research has demonstrated that polyphenol-rich extracts from wines and grapes have the ability to modulate mutagenesis and prevent tumor initiation and promotion [46]. Gronbaek et al. demonstrated that regular and moderate consumption of red wine is associated with a 22% decreased risk of cancer [60]. Various reports have demonstrated the anticancer action of red wine polyphenols in animal models. For example, tumoral C26 growth was significantly reduced with red wine polyphenols in BALB/c mice [27]. In this study, red wine extract (RWE) decreased tumor vascularization and the expression of proangiogenic factors including vascular endothelial growth factor (VEGF), metalloproteinases (MMP-2, MMP-9), and cyclo-oxygenase-2 (COX-2) proteins (Figure 2). The inhibition of cell proliferation with the expression of p21, an inhibitor of the cell cycle, and the expression of tumor suppressor genes, including p16INK4A, p53, and p73, was observed. RWE is able to act on the development of colorectal cancer through the prevention of aberrant crypt foci (ACF) (Figure 2). In a preclinical study on male CF-1 mice developing intestine polyp preneoplasia with azoxymethane (AOM) injection [61], we showed that mice receiving red wine extract in the diet, at a concentration of 500 mg/kg for 6 weeks, reduced the total number of AFCs per centimeter of colon as compared to the control group [26]. This result is interesting because the lower part of the colon is often associated with rectal cancer. Very recently, this study was reinforced by a report showing that dried red wine, pomegranate extract, and α-tocopherol added at one dose to cured meat, and the withdrawal of sodium erythorbate, significantly decreased the number of precancerous lesions (mucin-depleted foci, MDF) per colon in rats [62]. However, white grape and rosemary extracts, which do not have the same polyphenol composition, did not. The molecular mechanism involved seems to be the suppression of fecal excretion of nitrosyl iron [62].

Other targets that have been thoroughly described in the occurrence of colorectal cancer are the enzymes involved in arachidonic acid metabolism (i.e., cyclooxygenase and lipoxygenase). The latter is inhibited in the presence of grape seed extract or red wine polyphenolic compounds in various colorectal cancer cell lines or hepatocarcinoma cells [63].

Therefore, the present findings provide in vivo evidence for the antiangiogenic, antiproliferative, and proapoptotic effects of red wine polyphenols, associated with an effective inhibition of colon carcinoma tumor growth in mice. Red wine polyphenols target several key processes for tumorigenesis, supporting their role as potential chemopreventive agents against cancer.

### 2.3. Wine and Prostate Cancer

Prostate cancer (PCa) is the fourth most common form of cancer occurring worldwide. A total of 1.28 million cases each year are recorded [28]. There are well-established risk factors for PCa, such as family history [64,65], hereditary genes [66], racial and ethnic background, and age. Also, a wide variety of exogenous, environmental, and lifestyle factors have been shown to impact the risk of PCa development and progression. A study has shown a significant dose–response relationship between the level of alcohol intake and the risk of PCa [67]. Despite an association between alcohol intake and PCa risk, the effect of wine consumption on PCa risk is not yet fully understood.

According to the meta-analysis undertaken by Vartolomei et al. on 17 studies (611,169 subjects), there is an antagonist effect, such that moderate white wine consumption increases the risk of PCa, whereas moderate red wine consumption had a protective role against PCa [68]. This difference can be explained by the anticarcinogenic effect of polyphenols that are mainly found in red wine, which may balance any other negative or harmful effects, and by the bioactivity of the polyphenols present in red wine.

A recent study with a grape skin extract (MSKE), which is derived from muscadine red grapes, highlights a molecular mechanism involving an activation of unfolded protein response (UPR)-mediated autophagy and the subsequently mediated apoptosis [69]. The existing cross-talk between autophagy and apoptosis has been considered a key factor in the development and treatment of cancer [70]. MSKE induced apoptosis via the up-regulation of endoplasmic reticulum (ER) stress-driven caspase-3, -7, and -12. MSKE also prompted the down-regulation of antiapoptotic and survival proteins, such as annexin A4 (ANXA4), a member of the Ca^2+^-regulated and phospholipid-binding annexin superfamily, which is regularly increased in many cancer types [71,72,73].

Colorectal and prostate cancers are not the only ones that can be affected by the action of wine polyphenol extracts; many other studies have shown an induction of apoptosis and a modulation of the protein that controls either oxidative stress or the cell cycle on many models, such as breast cancer [74] and leukemias [75].

In a similar manner to the different compounds that compose it, the wine extracts can modulate the activation of the kinase cascades induced by many activators. Resveratrol, a major phenolic component, can inhibit the mitogen-activated protein kinase (MAPK) pathway activation, which is mediated by various promoters [76,77]. For example, in vitro, resveratrol inhibits phorbol-12-myristate-13-acetate (PMA)-mediated activation of c-Jun N-terminal kinase (JNK) [78]. In vivo, pretreatment of the dorsal skin of female mice with resveratrol decreases the phosphorylation of extracellular signal-regulated protein kinase (ERK), as well as the catalytic activity of ERK and p38 MAPK, which are stimulated by various stimuli [77,79,80]. Lee et al. reported that red wine extract inhibited 12-O-tetradecanoylphorbol-13-acetate (TPA)-induced transformation of JB6 promotion-sensitive mouse skin epidermal cells. The activation of activator protein-1 (AP1) and nuclear factor-kappa B (NFκB), induced by TPA, was dose-dependently inhibited by RWE treatment [81]. Consequently, by its blocking action on the stimulus-mediated MAPK pathway activation, red wine extract could possess an antitumor-promoting property.

### 2.4. Other Beneficial Properties of Wine in Cancer

These polyphenol extracts could both serve as sources of new bioactive compounds and also reduce the deleterious effects of certain chemotherapies. A study on various cancers has shown that a red wine extract lowered the side-effect toxicity of cisplatin treatment [82]. In the same way, a grape seed extract has been shown to protect intestinal epithelial cells prior to damage induced by 5-fluorouracil (5-FU) in female Dark Agouti rats [81]. These females were gavaged with 1 mL of polyphenol extract (400 mg/kg) daily (day three to 11), and received 5-FU (150 mg/kg) by intraperitoneal injection on day nine to induce mucositis. Compared with 5-FU controls, polyphenol extract significantly reduced myeloperoxidase activity in the proximal jejunum and distal ileum, decreased qualitative histological scores of damage in the proximal jejunum, increased villus height in the proximal jejunum and distal ileum, and attenuated the 5-FU-induced reduction of mucosal thickness by 16% in the jejunum and 45% in the ileum [81].

More interestingly, red wine extract can present an additive effect, when combined with cyclophosphamide or with cisplatin, to inhibit the tumor growth in mice with Ca755 mammary carcinoma or with Guerin carcinoma [82].

In view of these results, many teams and firms then made lyophilized extracts enriched with polyphenols from grapevines. For example, Liofenol™ contains natural Gocciorosso red wine lyophilized extracts, is devoid of alcohol, and is composed of a variety of components, such as polyphenols [83]. This preparation reduces colon cancer cells with an increase of p53 and p21 protein expression. Moreover, the authors observed a strong induction of antioxidant response, with the activation of the transcriptional factor Nrf2, involved in redox homeostasis and differentiation, without altering tumor sensitivity to chemotherapy with Tam and etoposide [83].

## 3. Conclusions

Moderate wine consumption, part of the Mediterranean diet, has been associated with potential protective effects, not only on cardiovascular pathologies but also on several cancer types. These protective effects have been attributed to wine microconstituents, such as polyphenols, among which resveratrol has been the most widely studied so far. However, much effort has been extended to characterize and understand the complex composition of wines, in particular red wine, whose polyphenol content is highly dependent on winemaking processes, the type of vine, climate, country, and the age of the wine. We have notably provided evidence that the efficacy of red wine extracts in inhibiting colon cancer cell line proliferation depended on their polyphenolic content and composition. Indeed, taken individually, red wine polyphenols have been shown to limit several stages of tumorigenesis, supporting their chemopreventive properties; however, some polyphenols may have antagonist activities, thus limiting their beneficial effects. These aspects are of importance as not all preclinical trials in humans have shown a positive effect of wine extract consumption on the occurrence of cancers, which could be partly explained by the dose used and the composition of the extracts. Nonetheless, a great deal of evidence supports the bioactivity of red wine extracts in various cancer type models, both in vitro and in vivo, in animals. Compared to individual red wine chemical constituents, RWE has been able to target the most commonly deregulated signaling pathways in cancer and prevent some of the side effects of conventional chemotherapies. Hence, diet supplementation with RWE, which does not seem to present any toxicity, at least in animal models, would be valuable in chemoprevention strategies; however, much work remains to be done to discover how best to use it (e.g., identification of the most efficient combination of microcomponents for synergistic effects), which may benefit from taking into account individual genetic variability and metabolism.
